# Adult Pneumococcal Vaccination in Latin America: The Next Step Beyond Pediatric Pneumococcal Conjugate Vaccine

**DOI:** 10.7759/cureus.106582

**Published:** 2026-04-07

**Authors:** Esteban Zavaleta-Monestel, Sebastián Arguedas-Chacón, Jeaustin Mora-Jiménez, José Pablo Díaz-Madriz, Luis Guillermo Herrera-Jiménez

**Affiliations:** 1 Pharmacy, Hospital Clínica Bíblica, San José, CRI; 2 Research, Hospital Clínica Bíblica, San José, CRI; 3 Pharmacy, University of Costa Rica, San José, CRI

**Keywords:** adult immunization, invasive pneumococcal disease, latin america, pneumococcal vaccination, streptococcus pneumoniae

## Abstract

Pneumococcal disease remains an important cause of morbidity and mortality among adults, particularly older individuals and those with chronic medical conditions. While pediatric pneumococcal conjugate vaccine (PCV) programs have substantially reduced vaccine-type disease and generated indirect protection through herd effects, a persistent burden of pneumococcal infection continues to affect adult populations in Latin America. Demographic ageing, the growing prevalence of chronic diseases, immunosenescence, and shifts in circulating pneumococcal serotypes contribute to sustaining this residual risk despite successful pediatric vaccination strategies. As epidemiologic patterns evolve, prevention policies must also adapt. Direct vaccination of adults offers an opportunity to reduce severe pneumococcal disease, hospitalisations, and preventable deaths among high-risk populations. Integrating adult pneumococcal vaccination into existing healthcare platforms, including primary care visits, chronic disease management programs, and hospital discharge protocols, represents a feasible pathway for implementation in the region. Strengthening adult immunisation strategies should therefore be viewed as the logical next step in pneumococcal disease prevention in Latin America, complementing the substantial progress achieved through pediatric vaccination programs.

## Editorial

Pediatric pneumococcal conjugate vaccine (PCV) programmes have fundamentally altered the epidemiology of invasive pneumococcal disease (IPD) in Latin America. Sustained childhood immunisation has led to substantial reductions in vaccine-type disease and has generated considerable indirect protection among unvaccinated populations through herd effects [1].

However, despite more than a decade of widespread pediatric PCV implementation, severe pneumococcal disease continues to impose a substantial burden among adults. Pneumococcal pneumonia and invasive disease remain important causes of hospitalisation and mortality, particularly among older adults and individuals with chronic medical conditions [2]. This persistence does not undermine the success of pediatric immunisation programmes; rather, it reflects a structural shift in epidemiologic risk that current prevention policies have yet to address.

Adult pneumococcal infection is associated with considerable morbidity and mortality. Advanced age, together with chronic cardiometabolic, pulmonary, renal, and immunocompromising conditions, markedly increases susceptibility to invasive disease and adverse outcomes [3]. Beyond the acute episode, hospitalisation frequently requires intensive supportive care. Long-term mortality after community-acquired pneumonia remains elevated for years following hospitalisation, suggesting persistent inflammatory and cardiovascular sequelae that contribute to progressive vulnerability in older adults [4]. Preventing a single episode of severe pneumococcal disease may therefore avert a broader trajectory of clinical decline.

Regional epidemiological studies indicate that adults aged 60 years and older represent a growing proportion of IPD cases in Latin America, with case-fatality rates frequently exceeding 15-20% in this group [5]. At the same time, surveillance data show that an increasing share of adult disease is caused by serotypes not included in earlier PCV formulations [6]. These patterns illustrate that the remaining adult burden is not a failure of pediatric vaccination programmes but rather the expected epidemiological remainder once herd protection reaches its practical limits.

Indirect protection has well-documented benefits, but it also faces biological constraints. As vaccine-type serotypes decline, non-vaccine serotypes increasingly occupy available ecological niches, resulting in stabilisation rather than continued reduction of overall disease incidence [7]. Serotype replacement is an anticipated adaptive response within pneumococcal population dynamics. Consequently, reducing transmission among children cannot indefinitely prevent disease among biologically vulnerable adults when the circulating serotype composition continues to evolve [8].

**Figure 1:**
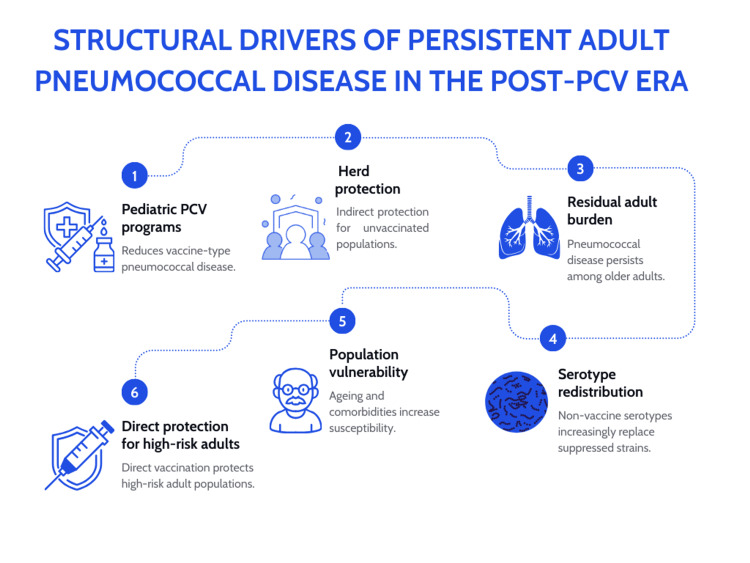
Structural drivers of persistent adult pneumococcal disease in the post-PCV era. Structural and epidemiological factors sustaining adult pneumococcal disease burden despite successful pediatric pneumococcal conjugate vaccination (PCV) programs, including demographic ageing, chronic disease prevalence, immunosenescence, and serotype redistribution. Source: The figure was manually designed by the authors using Canva (Canva Pty Ltd., Sydney, Australia) and does not contain generative AI-generated content.

Demographic changes amplify this vulnerability. Latin America is experiencing rapid population ageing alongside increasing prevalence of diabetes, chronic obstructive pulmonary disease, cardiovascular disease, and frailty syndromes, all of which independently elevate pneumococcal risk. Immunosenescence compounds this problem by weakening innate and adaptive immune responses, reducing pathogen clearance and increasing susceptibility to infection in older populations [9]. Together, these forces sustain a large pool of susceptible adults for whom indirect protection alone is insufficient. The structural drivers sustaining this persistent adult burden in the post-PCV era are summarised in Figure 1.

Pediatric PCV programmes substantially reduce vaccine-type disease and transmission through herd protection. However, demographic ageing, immunosenescence, chronic disease clustering, and serotype redistribution sustain a residual burden of pneumococcal disease among adults. These structural pressures explain why indirect protection alone cannot eliminate adult disease and highlight the rationale for adult pneumococcal vaccination as the next phase of prevention policy in Latin America.

If epidemiologic risk has shifted toward older adults, prevention strategies must adapt accordingly. An approach involves age-based adult pneumococcal vaccination beginning at 60 or 65 years, complemented by risk-based vaccination for individuals with major chronic conditions. Unlike herd protection, which depends on reducing transmission in younger populations, adult vaccination provides direct protection against invasive disease and severe pneumonia.

Recent expansions of adult conjugate vaccine recommendations in several countries reflect growing recognition that persistent adult burden warrants direct immunological protection [10]. In Latin America, the integration of adult vaccination into existing healthcare platforms offers a feasible pathway for implementation. Vaccination could be embedded into hospital discharge protocols, chronic disease clinics, and primary care encounters, facilitating identification of high-risk individuals without requiring new infrastructure.

Budgetary constraints are often cited as a barrier to expanding adult vaccination programmes in the region. Yet as the population aged 60 years and older grows, the burden of hospitalisations and preventable deaths will rise regardless of vaccination policy. A substantial proportion of this burden remains preventable through timely adult immunisation, making investment in direct protection an economically rational response [10].

Pediatric PCV programmes remain indispensable for pneumococcal disease control. Nevertheless, demographic ageing, immunosenescence, chronic disease burden, and serotype redistribution have shifted the epidemiologic landscape toward older adults. Indirect protection alone is unlikely to eliminate preventable pneumococcal disease in this population. Adult pneumococcal vaccination should therefore be viewed not as an optional extension of pediatric programmes but as the logical next phase of pneumococcal prevention policy in Latin America.
